# Determinants of vitamin D status in physically active elderly in the Netherlands

**DOI:** 10.1007/s00394-018-1856-1

**Published:** 2018-12-06

**Authors:** D. S. M. ten Haaf, M. G. J. Balvers, S. Timmers, T. M. H. Eijsvogels, M. T. E. Hopman, J. M. T. Klein Gunnewiek

**Affiliations:** 1 Radboud Institute for Health Sciences, Department of Physiology, Radboud University Nijmegen Medical Centre, Nijmegen, The Netherlands; 2 Clinical Chemistry and Haematology Laboratory, Gelderse Vallei Hospital, P.O. Box 9025, 6710 HN Ede, The Netherlands; 3 Division of Human Nutrition, Wageningen University, Wageningen, The Netherlands; 4 Human and Animal Physiology, Wageningen University, Wageningen, The Netherlands

**Keywords:** Vitamin D status, 25(OH)D, Elderly, Determinant, Physical activity, Dietary intake

## Abstract

**Purpose:**

Vitamin D deficiencies are common in elderly, which increases the risk for, e.g., bone fractures. Identification of determinants of vitamin D status may provide leads for specific deficiency prevention strategies. Although determinants of vitamin D status have been studied in various populations, this has not been examined in elderly that have a physically active lifestyle.

**Methods:**

Vitamin D status of 450 physically active elderly who do not use vitamin D supplements was determined and information on possible determinants (demographic, dietary intake and physical activity) was collected around a prolonged four day walking event in July and analyzed in linear regression models.

**Results:**

The average summertime serum 25(OH)D concentration was 88.8 ± 22.4 nmol/L. Only 2% of the participants had a 25(OH)D concentration below 50 nmol/L. Dietary intake of vitamin D was 4.0 ± 1.9 µg/day, and the participants spent 12.4 ± 8.6 h/week on outdoor activities. In the multivariate model, lower age (= − 0.48, 95% CI − 0.80 to − 0.16), lower BMI (= − 0.86, 95% CI − 1.62 to − 0.10), being a moderate to high drinker versus a non-drinker (= 7.97, 95% CI 0.43–15.51) and more outdoor physical activity (= 0.25, 95% CI 0.01–0.50) were significantly associated with higher 25(OH)D concentrations.

**Conclusions:**

In physically active elderly, vitamin D status was very high in summertime, with few deficiencies, suggesting that elderly with a physical active lifestyle might not necessarily need supplements during the summer period. Lower age, lower BMI, higher alcohol intake and more outdoor physical activity had a significant association with vitamin D status.

## Introduction

Vitamin D is an essential micronutrient that has several functions, such as the formation of bone tissue and absorption of calcium from the gastrointestinal tract [1, 2]. The most important source of vitamin D is the skin, which can produce vitamin D from 7-dehydrocholesterol during exposure to ultraviolet (UV) radiation [3]. The rate of cutaneous vitamin D synthesis is reduced in elderly, and therefore they are at risk for vitamin D deficiencies [4]. For instance, in the Netherlands, about 50% of community-dwelling elderly has a vitamin D deficiency [5], which has led to standard supplementation guidelines for elderly [6–8]. However, blood concentrations of 25-hydroxy vitamin D (25(OH)D), the accepted vitamin D status marker [9], can vary considerably between persons, even between persons that appear to receive the same daily dose of vitamin D [10]. This suggests that other factors affect concentrations of 25(OH)D and that the current generalized vitamin D supplementation practices may be inadequate in certain cases. Moreover, based on the age-dependent decline in cutaneous vitamin D synthesis, it may be expected that vitamin D status is lower in subgroups of higher age, but this has not been demonstrated before. A better understanding of the determinants of vitamin D status is therefore required to improve vitamin D status at both the individual as well as the population level.

In recent years, several publications have aimed to identify potential determinants of vitamin D status, such as use of supplements, age and lifestyle factors [5, 11–16]. However, these studies have several limitations, amongst others a limited physical activity range of the participants. Especially, knowledge on vitamin D status and its determinants in physically active elderly is lacking.

In the present study, the vitamin D status is investigated in different age subgroups in physically active elderly aged 65–93 year who do not use vitamin D supplements. In addition, determinants that contribute to vitamin D status were explored. We hypothesized that vitamin D status is relatively high in physically active elderly, and that dietary intake and outdoor physical activity are significant contributors to vitamin D status.

## Materials and methods

### Study population

Participants of the 4 Days Marches of 2015 or 2016, an annual 4 day walking event in the Netherlands that takes place in July, were recruited via newsletters and internet advertisements. Participants had to be 65 year or older and Caucasian. The study adhered to the Declaration of Helsinki. The Medical Ethical Committee of the Radboud University Medical Center approved the study (study-id: NL36743.091.11), and all participants gave written informed consent prior to participation.

### Study design

During this cross-sectional study, participants filled in two online questionnaires. The first questionnaire assessed demographic characteristics (sex, age, ethnicity, body weight and height and smoking), use of supplements and habitual physical activity levels with the validated SQUASH questionnaire [17]. The second questionnaire was a validated food frequency questionnaire about their habitual dietary intake [18, 19].Furthermore, participants visited our field laboratory at the event location 1 or 2 days prior to the first walking day to collect a venous blood sample of 3.5 ml.

### Analysis of blood vitamin D concentrations

Venous blood was drawn from the antecubital vein in Vacutainer collection tubes (Becton Dickinson, Vianen, the Netherlands) and was allowed to clot for at least 30 min at room temperature. Within 4 h after collection, the blood was centrifuged and serum was stored at − 80 °C until further analysis. Serum 25(OH)D3 concentrations were determined using a commercially available kit with high-performance liquid chromatography coupled to ultraviolet detection (HPLC-UV; Chromsystems Instruments & Chemicals GmbH, Gräfelfing, Germany) for samples collected in 2015 (*n* = 378), or a method using liquid chromatography coupled to tandem mass spectrometry detection (LC-MS/MS; Waters Chromatography B.V., Etten-Leur, the Netherlands) for samples collected in 2016 (*n* = 72). Briefly, both methods consisted of a protein precipitation step and solid phase extraction prior to analysis on the HPLC-UV or LC-MS/MS system. Calibrators from the same source (Chromsystems) were used on both systems. Quality control samples at different concentrations were included in each analytical batch to monitor the quality of the analysis. All analyses were performed in the Clinical Chemistry and Haematology Laboratory of Gelderse Vallei Hospital (Ede, the Netherlands) by trained technicians using standard operating procedures. A previously performed direct comparison of the in-house HPLC and LC-MS/MS methods revealed that 25(OH)D concentrations obtained with the LC-MS/MS method were on average 10% higher than the HPLC method results (internal method validation report, unpublished data); therefore, a correction factor of − 10% for the LC-MS/MS values was applied to align the 25(OH)D data prior to further statistical analyses.

### Physical activity

Physical activity was assessed by the validated Short Questionnaire to Assess Health enhancing physical activity (SQUASH) [17]. SQUASH estimates habitual physical activity during a normal week over the past month. Questions include the type, duration and frequency of activities. The total amount of physical activity in hours per week (hr/wk) was calculated [20]. Participants were excluded if questionnaires were incomplete and when the total minutes of activity per day exceeded 960 min [17]. We incorporated domestic work activities, leisure time activities and sports to assess activities of daily living (i.e., total physical activity). Individual activities were categorized as “outdoor” based on discussion with experts that are familiar with the physical activity habits in the Netherlands. Hours per week spent on outdoor leisure time activities and sports activities were calculated.

### Dietary assessment

An online validated 180-item semi-quantitative Food Frequency Questionnaire (FFQ) was used to assess habitual daily energy intake, vitamin D intake and alcohol consumption [18, 19]. The FFQ reference period was 1 month, and portion sizes were estimated using standard portions [21]. Nutritional intake was calculated using the Dutch Food Composition Database of 2010 [22]. Some participants were not able to fill in the online questionnaires and dieticians assessed their daily dietary intake with two 24-h recalls (*n* = 30). 2 days were randomized over the week with the restriction that no participant was assigned two identical week days (e.g., two Mondays) or two weekend days (e.g., Saturday and Sunday). The mean of both days was considered to represent their common eating pattern. Alcohol consumption was derived in gram per day of pure alcohol. Based on the alcoholic one drink-equivalent of 14 g of pure alcohol and the American guidelines [23], we divided the participants into non-drinkers, low drinkers, moderate drinkers and high drinkers. A non-drinker was defined as 0.0–2.0 gram of alcohol per day which is equivalent to zero to maximally one drink per week. A low drinker was defined as 2.06–20.86 gram for females and 2.06–34.86 gram for males, which is equivalent to ≥ 1 glass per week to 1.5 or 2.5 glasses per day for females and males, respectively. A moderate drinker was defined as ≥ 1.5 glasses to 3.5 glasses per day for females (20.87–48.86 gram) and ≥ 2.5 glasses to 4.5 glasses per day for males (34.87–63.0 gram). A high drinker was defined as ≥ 3.5 glasses per day for females (≥ 48.87 gram) and ≥ 4.5 glasses per day for males (≥ 63.06gram).

### Statistical analysis

The statistical analyses were performed using SPSS 22 software (IBM SPSS Statistics for Windows, Version 22 IBM Corp., Armonk, NY, USA), with the level of significance set at *p* < 0.05 (two-sided). Participant characteristics were displayed as means ± SDs or as counts with percentages for categorical variables. The total group was divided in three age groups (65–74 year, 75–84 year and 85–93 year) and differences in serum 25(OH)D concentration and baseline characteristics were analyzed between age groups using one-way ANOVA, and using the Chi square test or Fisher’s exact test for categorical variables. Furthermore, after checking the assumptions for linear multiple regression, the associations between possible determinants (i.e., age, sex, BMI, smoking status, vitamin D intake via nutrition, alcohol intake and physical activity) and serum 25(OH)D concentration (nmol/L) were analyzed using univariate and multivariate linear regression model (forced entry method). To avoid large discrepancies in subgroup sizes, the moderate and high alcohol intake groups were merged.

## Results

### Population characteristics

**Fig. 1:**
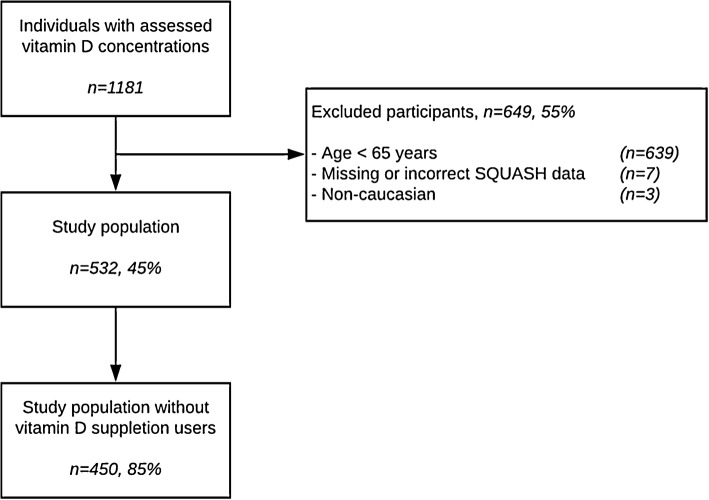
Flowchart for enrollment of the study population

**Fig. 2:**
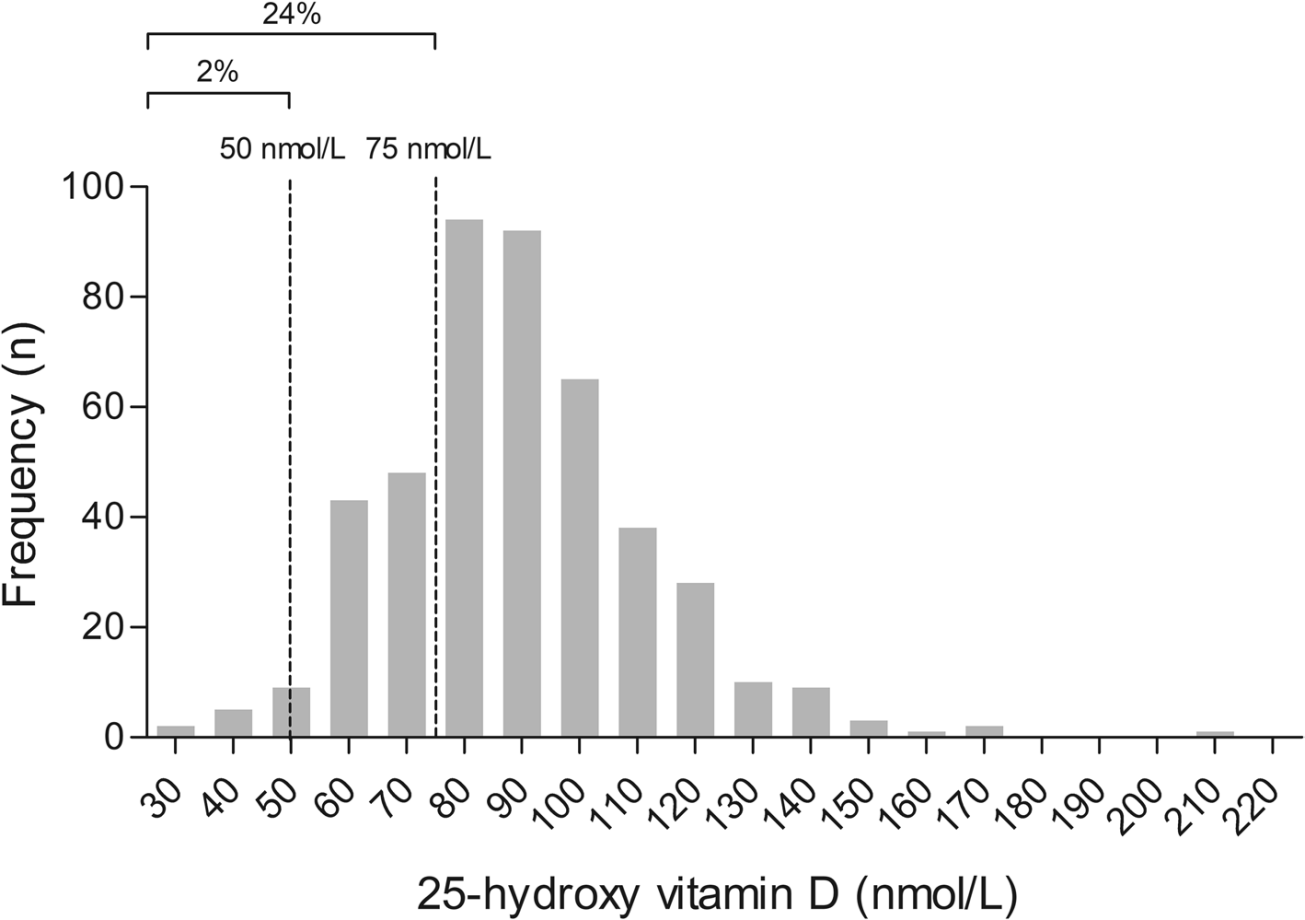
Frequency distribution of 25(OH)D concentrations (nmol/L) of 450 physically active elderly that do not use vitamin D supplements. Mean 25(OH)D concentrations was 88.8 ± 22.4 nmol/L. A total of 2% were below the threshold for 25(OH)D concentration of 50 nmol/L and 24% were below the 75 nmol/L threshold for 25(OH)D concentration. These findings suggests that elderly who are physically active are able to reach a good vitamin D status, with a low prevalence of deficiencies

We included 450 physically active elderly between the age of 65 and 93 in the present study (Fig. 1; Table 1). Seventy-eight percent of the participants were male, aged 71.9 ± 6.8 year and with a BMI of 25.0 ± 2.9 kg/m^2^. The mean serum 25(OH)D concentration in the summer was 88.8 ± 22.4 nmol/L, and serum 25(OH)D concentrations < 50 nmol/L and < 75 nmol/L were present in 2% and 24% of the population, respectively (Fig. 2; Table 1). The mean daily energy intake was 2264 ± 650 kcal for males and 1934 ± 463 kcal for females. The vitamin D intake via nutrition was 4.0 ± 1.9 µg/day, with 99% of the participants having an intake below the generally accepted recommendation of 20 µg/day [1, 24]. The participants spent 12.4 ± 8.9 h/week on outdoor activities.

### Serum 25(OH)D concentrations across 10 year age groups

Mean serum 25(OH)D concentrations were 91.0 ± 23.1 nmol/L, 84.1 ± 19.2 nmol/L and 77.8 ± 18.6 nmol/L for the age groups 65–74 year, 75–84 year and 85–93 year, respectively (Table 1). Although mean 25(OH)D values were not significantly different between the age subgroups, significantly more participants in the 85–93 year group had a serum 25(OH)D concentration ≥ 50 nmol/L, whereas less participants in this oldest age group had serum 25(OH)D concentration ≥ 75 nmol/L compared to the younger age groups. Moreover, sex, BMI, smoking, vitamin D intake via nutrition and alcohol intake did not differ between the age groups. Total physical activity (h/wk) was significantly higher in participants aged 65–74 year versus participants aged 75–84 year (*p* = 0.037). Participants aged 65–74 year performed more sports activities compared to participants aged 75–84 year (*p* = 0.007). Outdoor physical activities were not significantly different between age groups.

**Table 1:** Baseline characteristics of the participants that do not use vitamin D supplementation, stratified by 10 year age groups

Variable	Total *n* = 450	65–74 year *n* = 331	75–84 year *n* = 94	85–93 year *n* = 25	*p* value
Age, yr	71.9 ± 6.8	68.3 ± 2.7	80.6 ± 3.0	87.1 ± 1.9	< **0.001**
Male, *n* (%)	353 (78)	257 (78)	77 (82)	19 (76)	0.64*
BMI, kg/m^2^	25.0 ± 2.9	25.1 ± 2.9	24.9 ± 3.0	24.0 ± 2.2	0.13
Currently smoking, *n* (%)	19 (4)	18 (6)	1 (1)	0 (0)	0.13^‡^
**Vitamin D status**					
25(OH)D, nmol/L	88.8 ± 22.4	91.0 ± 23.1	84.1 ± 19.2	77.8 ± 18.6	0.092
25(OH)D ≥ 50 nmol/L, *n* (%)	441 (98)	324 (98)	92 (98)	25 (100)	< 0.001*
25(OH)D ≥ 75 nmol/L, *n* (%)	343 (76)	268 (81)	62 (66)	13 (52)	< 0.001*
**Dietary intake**					
Vitamin D via nutrition, µg	4.0 ± 1.9	4.1 ± 1.7	4.1 ± 2.4	3.2 ± 2.7	0.09
Alcohol, g/d	14.4 ± 14.6	15.2 ± 14.8	12.4 ± 14.0	10.7 ± 13.1	0.13
Non-drinker, *n* (%)	99 (22)	64 (19)	26 (28)	9 (36)	0.14‡
Low drinker, *n* (%)	289 (64)	223 (67)	53 (56)	13 (52)	
Moderate drinker, *n* (%)	45 (10)	38 (12)	5 (5)	2 (8)	
High drinker, *n* (%)	7 (2)	6 (2)	1 (1)	0 (0)	
**Total physical activity**					
Total physical activities, hr/wk	29.1 ± 16.4	30.4 ± 16.8	25.6 ± 14.3	25.3 ± 15.8	**0.021**
Domestic work activities, hr/wk	10.2 ± 10.7	10.3 ± 10.9	10.0 ± 10.7	8.6 ± 7.5	0.73
Leisure time activities, hr/wk	13.1 ± 9.4	13.6 ± 9.4	11.6 ± 7.8	13.2 ± 13.1	0.20
Sports activities, hr/wk	5.7 ± 6.1	6.3 ± 6.1	4.2 ± 5.1	3.5 ± 7.8	**0.002**
**Outdoor physical activity**					
Total physical activities outdoor, hr/wk	12.4 ± 8.6	12.8 ± 8.8	11.3 ± 7.6	10.4 ± 8.8	0.15
Leisure time activities outdoor, hr/wk	11.0 ± 7.9	11.4 ± 8.1	10.2 ± 7.2	10.2 ± 8.8	0.39
Sports activities outdoor, hr/wk	1.2 ± 2.9	1.3 ± 2.8	1.2 ± 3.4	0.2 ± 0.8	0.16

Data are presented as mean ± SD or number (percentage) of participants

Bold values indicate *β* with *p* value < 0.05

*BMI* body mass index, *25(OH)D* 25-hydroxy vitamin D

*Derived by Chi square test

^‡^Derived by Fisher’s exact test

### Determinants of serum 25(OH)D concentration

Lower age (*p* = 0.001), being a low or moderate to high drinker compared to a non-drinker (*p* = 0.011, *p* = 0.010, respectively) and more outdoor physical activity (*p* = 0.023) were associated with a higher serum 25(OH)D concentration in the univariate analysis, whereas sex, BMI, smoking and dietary vitamin D intake were not associated with serum 25(OH)D concentration (Table 2). In the multivariate model with correction for all variables, lower age (*p* = 0.003), lower BMI (*p* = 0.026), being a moderate to high drinker compared to a non-drinker (*p* = 0.038) and more outdoor physical activity (*p* = 0.046) were associated with a higher vitamin D status (Table 2). In total, these variables explained 5.9% of the variation of the serum 25(OH)D concentration. The assumptions of linear regression were met.

**Table 2:** Associations between demographic and lifestyle factors (sex, age, BMI, smoking, vitamin D via nutrition, alcohol consumption and physical activity) and 25(OH)D

	25(OH)D, nmol/L	
	Univariate *β* (95% CI)	Multivariate *β* (95% CI)*
**Age**, yr	− **0.54 (**− **0.84 to** − **0.23)**	− **0.48 (**− **0.80 to** − **0.16)**
**Sex** ^§^		
Male (ref)	1.00	1.00
Female	− 0.60 (− 5.66 to 4.46)	− 2.39 (− 7.83 to 3.06)
**BMI**, kg/m^2^	− 0.70 (− 1.43 to 0.03)	− **0.86 (**− **1.62 to** − **0.10)**
**Smoking** ^§^		
Non-smoker (ref)	1.00	1.00
Current smoker	1.34 (− 8.96 to 11.65)	− 1.24 (− 11.49 to 9.02)
Vitamin D via nutrition, µg	0.71 (− 0.40 to 1.82)	0.21 (− 0.93 to 1.35)
**Alcohol** ^§^		
Non-drinker (ref)	**1.00**	1.00
Low drinker	**6.41 (1.49–11.33)**	5.10 (− 0.15 to 10.36)
Moderate to high drinker	**9.70 (2.33–17.08)**	**7.97 (0.43–15.51)**
**Total physical activities outdoor**, hr/wk	**0.28 (0.04–0.52)**	**0.25 (0.01–0.50)**

Data were analyzed using linear regression with 25-hydroxy vitamin D (nmol/L) as the dependent variable

Bold values indicate *β* with *p* value < 0.05

*BMI* body mass index, *25(OH)D* 25-hydroxy vitamin D

*Adjusted for all variables shown in the table

^§^Categorical variable in which we indicated one option as the constant against which other options were compared

## Discussion

In the present study, the vitamin D status and its determinants were investigated in a group of physically active elderly in the summertime. The main findings were that physically active elderly who do not take supplements have high average 25(OH)D blood concentrations in the summer, with only ~ 2% of the population demonstrating a 25(OH)D concentration < 50 nmol/L. Dietary intake of vitamin D did not significantly contribute to vitamin D status, whereas lower age, lower BMI, higher alcohol intake and more outdoor physical activity were significantly associated with a higher vitamin D status in the multivariate model.

The average vitamin D status of 88.8 nmol/L in elderly aged 65–93 years, determined in July in the Netherlands, is substantially higher than reported in comparable studies. Brouwer-Brolsma et al. investigated vitamin D status in community-dwelling elderly aged ≥ 65 year, and reported a mean 25(OH)D concentration of 70 nmol/L in blood samples that were collected in July [5]. Moreover, in our population, only 2% had a blood 25(OH)D value of < 50 nmol/L, whereas Brouwer-Brolsma reported that 37% of the population had a blood 25(OH)D value < 50 nmol/L. Furthermore, van Dam et al. reported a mean 25(OH)D concentration of 61.3 nmol/L in the summer months with 33.7% < 50 nmol/L in an elderly population with a mean age of 69 year [12]. The dietary intake of vitamin D in the current study (4.0 ± 1.9 µg/day) is comparable to what is found previously by Brouwer-Brolsma (~ 4.0–4.5 µg/day) [5], and therefore it is unlikely that dietary intake explains the differences in vitamin D status between the study populations. A more plausible explanation for the higher average 25(OH)D concentration in the present study is that our population spent more time on outdoor physical activity. Previous studies have shown that (outdoor) physical activity is associated with a higher vitamin D status [13, 15]. In the current study, elderly spent on average 12.4 h/week on outdoor activities compared to an average < 7 h/week as reported by Van Dam [12]. Therefore, in all age categories (65–74 year, 75–84 year and 85–93 year), the substantially better vitamin D status in physically active elderly may be explained by higher levels of outdoor physical activity. This suggests that despite the age-related lower rate of cutaneous vitamin D synthesis [4], a high level of outdoor physical activity can compensate for this. Another explanation for the high vitamin D status in this population is the relative low BMI. A high BMI and/or adiposity is associated with a lower vitamin D status or response to supplementation, which is explained by volumetric dilution and/or sequestration in the adipose tissue [6, 12, 25–27]. Our group of physically active elderly had a mean BMI 25.0 ± 2.9 kg/m^2^, compared to a mean BMI of 27.5 ± 4.3 kg/m^2^and 26.8 ± 3.6 kg/m^2^ that was reported for Dutch elderly [5]. Possibly, the high level of (outdoor) physical activity may lead to a high vitamin D status through exposure to UV light as well as lowering the BMI.

Generally, elderly are considered a group at risk for vitamin D deficiencies, which has led to generalized vitamin D supplementation guidelines for elderly [2, 6]. Although we observed that significantly more elderly between 65 and 84 years had a 25(OH)D value ≥ 75 nmol/L compared to the 85–93 year group, the vitamin D status in the entire population is good considering that only 2% of the population had a blood 25(OH)D value < 50 nmol/L. These observations put general vitamin D supplementation guidelines to question, as it shows that physically active elderly seem to reach a sufficient vitamin D status without supplementation, at least in the summertime. It is important to note that we did not measure vitamin D status in winter months. Brouwer-Brolsma investigated the year time fluctuation of vitamin D status in elderly and reported a mean value of ~ 42 nmol/L in January as the lowest value, and ~ 70 nmol/L as the highest mean in July [5]. If this finding is extrapolated to our population and 30 nmol/L is subtracted from the summer values, the mean 25(OH)D value would be > 55 nmol/L in the winter, with 34% < 50 nmol/L and 8% < 30 nmol/L. A follow-up evaluation in the winter would be useful to determine to what extent 25(OH)D values will drop in the winter months in physically active elderly who in general remain physically active in winter months as well [5, 12]. The vitamin D status in physically active elderly is high in summertime, which suggests that vitamin D supplementation strategy should take lifestyle factors into account, such as outdoor physical activity, leading to a more personalized and targeted supplementation.

In both the univariate and the multivariate models, age, BMI and outdoor physical activity were associated with 25(OH)D concentrations. These results are in agreement with what has been reported in literature for adults and (community-dwelling)elderly [5, 12, 13, 15, 28], where negative associations were found between age, BMI and vitamin D status, and positive associations were found between physical activity and vitamin D status.

To our surprise, alcohol intake appeared as a significant contributor to vitamin D status in the multivariate regression model. A positive association between moderate alcohol consumption and vitamin D status has been reported in the literature before [29]. The average alcohol consumption in our population was 14.4 gr/day, and ranged between zero consumption up to 79.3 gr/day, meaning that the population contained non-drinkers, low, moderate and some high drinkers. Van Grootheest et al. observed a positive correlation between both moderate and high alcohol consumption and 25(OH)D blood levels in a healthy adult population in the Netherlands [25]. Similar associations were observed in a German and Finnish population of (elderly) adults [30, 31]. These findings have not been discussed extensively and their relevance for humans is as yet not known. It is possible that the association is explained by drinking outdoor rather than the alcohol itself. Considering that alcohol may also be consumed during, e.g., dinner or later in the evening (when UV-based vitamin D synthesis is no longer active) we believe that outdoor drinking certainly does not fully explain the association. In addition, literature suggests that alcohol itself may alter vitamin D metabolism. Experiments with female rats have demonstrated that chronic ethanol consumption leads to reduced renal CYP27B1 expression, with subsequent lower concentrations of 1,25-dihydroxy vitamin D (1,25(OH)_2_D, the active vitamin D metabolite), and higher 25(OH)D blood concentrations [32]. It is relevant to know whether the same occurs in humans, as this may lead to overestimation of vitamin D status, while the levels of the active vitamin D metabolite may in fact be decreased. Thus, more research is needed to determine whether the observed positive association between vitamin D status and alcohol intake in humans can be explained by altered vitamin D metabolism.

A limitation of the current study is that our questionnaire did not specifically determine the level of outdoor physical activity and exposure to UV radiation. However, we included participants who were training for a multi-day long-distance walking event and therefore most physical activity was performed outside. Furthermore, all vitamin D data were collected within 48 h, which enabled us to assess determinants of vitamin D status without seasonal effects in vitamin D concentrations. A potential problem of this approach is that we assessed vitamin D status in summer only, and we do not know to what extent these values decrease in winter months.

In conclusion, this study demonstrates that physically active elderly without any supplements have a good vitamin D status in the summer with a low prevalence of deficiencies. From the explored potential determinants of vitamin D status, age, BMI, alcohol intake, and outdoor physical activity contributed significantly to vitamin D status. This report shows that current generalized supplementation recommendations for elderly might lead to unnecessary supplementation in physically active subpopulations in the summer. More research is needed to understand the observed association between alcohol intake and vitamin D status.
